# The −*3279C*>*A* and −*924A*>*G polymorphisms* in the *FOXP3* Gene Are Associated With Viral Load and Liver Enzyme Levels in Patients With Chronic Viral Liver Diseases

**DOI:** 10.3389/fimmu.2018.02014

**Published:** 2018-09-04

**Authors:** Leonn M. S. Pereira, Ednelza da Silva Graça Amoras, Simone R. S. da Silva Conde, Sâmia Demachki, Jaqueline C. Monteiro, Rosimar N. Martins-Feitosa, Andrea N. M. R. da Silva, Ricardo Ishak, Antonio C. R. Vallinoto

**Affiliations:** ^1^Laboratório de Virologia, Instituto de Ciências Biológicas Universidade Federal do Pará, Belém, Brazil; ^2^Faculdade de Medicina, Instituto de Ciências da Saúde, Universidade Federal do Pará, Belém, Brazil

**Keywords:** immune regulation, regulatory T lymphocytes, FOXP3, SNPs, chronic viral liver diseases

## Abstract

The transcription factor FOXP3 is an essential marker of the development and activation of regulatory T cells (Tregs), which are cells specialized in the regulation and normal tolerance of the immune response. In the context of chronic viral liver diseases, Tregs participate in the maintenance of infections by promoting histopathological control and favor the immune escape of viral agents by suppressing the antiviral response. Single nucleotide polymorphisms (SNPs) may influence the function of FOXP3 in a number of pathological conditions. The present study sought to evaluate the influence of SNPs in the *FOXP3* gene promoter region in patients with chronic viral liver diseases. Three SNPs (−*3279C*>*A*, −*2383C*>*T*, and −*924A*>*G*) were analyzed in groups of patients with chronic hepatitis C (CHC), active chronic hepatitis B (CHB-A), inactive chronic hepatitis B (CHB-I), and a healthy control group (CG) using real-time PCR. The frequencies of the polymorphic variants were compared between groups and correlated with liver histopathological characteristics and enzyme levels [i.e., alanine aminotransferase (ALT), aspartate aminotransferase (AST) and gamma-glutamyl transpeptidase (GGT)] obtained via biopsy and from the clinical records of the participating patients, respectively. For the −*2338C*>*T* SNP, no significant differences were found in the frequencies of variants between groups or in the histological findings. Significant associations between the polymorphisms and the CHB-I group were not established. The −*3279C*>*A* SNP was associated with altered viral loads (log_10_) and GGT levels in CHC patients with advanced stages of inflammatory activity and liver fibrosis. The −*924A*>*G* SNP was associated with altered viral loads (log_10_) and liver enzyme levels among CHB-A patients with milder inflammation and fibrosis. However, the frequencies of the −*3279C*>*A* and −*924A*>*G* polymorphisms were not directly associated with the histopathological profiles of the analyzed patients. These polymorphic variants may influence hepatic function in patients with chronic viral liver diseases but are not directly associated with the establishment of the degree of inflammatory activity and liver fibrosis.

## Introduction

Previous studies have shown that a sustained helper T and cytotoxic T lymphocyte response is closely associated with the disease courses of hepatitis B and C infections and that these cells are crucial for successful infection control (1–3). A stronger immune response with multiple targeted epitopes is expected during the acute phase of infection; in the chronic phase, in contrast, the immune response is less effective as a result of escape mutation(s) and/or T cell exhaustion due to sustained antigenic stimulation (4–6).

However, the continued actions of these cells in liver tissue can cause damage and autoimmune reactions, especially in the context of chronic viral infection. Therefore, many regulatory mechanisms of the immune system control specific immune responses against viruses to avoid these problems (7–9). The liver itself, which is constantly exposed to antigenic stimulation, has acquired specialized mechanisms and cells that mediate immunotolerance properties and prevent excessive activation of the immune response (10–12).

In this context, there is strong evidence for the involvement of a specialized set of cells called regulatory T cells (Tregs) in the regulation of both the liver immune response and tolerance (13). During chronic hepatitis B infection, the number of Tregs has been reported to be elevated in hepatic tissue and blood in the host and is correlated with the serological status of the infection and the patient's clinical condition (14–17). In chronic hepatitis C infection, different Treg subpopulations protect the host against tissue damage, and these cells are correlated with the degree of hepatic inflammation (18).

During hepatitis B and C infection, the numerical increase in Tregs occurs due to constant tissue immunological activation (19). Viral agents utilize this robust regulatory activity (20) but also modulate it by directly influencing Treg activation, which is a known escape mechanism of the antiviral response (21). Treg activation may also influence the progression of liver disease due to the maintenance of fibrogenesis and inflammatory tissue activity (22–24).

One key marker of Tregs is the transcription factor FOXP3 (Forkhead box P3). FOXP3 is a 47-kDa protein 431 amino acids in length (25), whose function is strictly related to the development of different Treg lines and the maintenance of immunoregulation in different pathological conditions that are both autoimmune and infectious in nature (26).

Expression of the *FOXP3* gene can be triggered by distinct biological stimuli, such as the presentation of host autoantigens to T cells (27, 28), or by alternative pathways that include interactions with normal microbiota metabolites (29) and the host cytokines transforming growth factor (TGF)-β and interleukin (IL)-10 (30). The activated FOXP3 protein prevents the interaction of nuclear factor of activated T cells (NFAT) and the nuclear factor kappa B (NF-kβ) transcriptional factors with genes associated with the expression of immune response-related cytokines [e.g., IL-4 and interferon (IFN)-γ] (31, 32). However, it favors the expression of genes that confer a regulatory phenotype [i.e., CD25, CTLA-4 and glucocorticoid-induced TNFR-related protein (GITR)] (33). Thus, FOXP3 is able to activate natural Treg lines or convert non-natural lines to suppressor cells (34).

The biological significance of single nucleotide polymorphisms (SNPs) in preserving the immune response role of FOXP3, and consequently in disease susceptibility, has previously been investigated (26). The promoter region of the *FOXP3* gene may harbor relevant SNPs, as it is involved in the regulation of gene expression and Treg activation (35, 36). Among these SNPs, the −*2383C* > *T* (rs3761549), −*3279C* > *A* (rs3761548) and −*924A* > *G* (rs2232365) SNPs are functionally well-defined and are distinguished by the relevance of studies concerning them.

The −*2383C*>*T* polymorphism was functionally characterized by Inoue et al. who suggested that the ^*^C allele altered the binding site of the transcription factor Ying Yang 1 (YY1) to the gene (37). The CC genotype decreases the regulatory function by increasing the activity of self-reactive T cells, causing severe thyroid tissue destruction in patients with Hashimoto's disease (HD) (38). Additional published studies have also associated this polymorphism with endometriosis, regardless of the disease stage (39), psoriasis (40), food allergies (41), and systemic lupus erythematosus (SLE) (42). Only one prior study has evaluated the influence of SNP −*2383C*>*T* on HBV and HCV infections; the presence of the polymorphism was associated with hepatocellular carcinogenesis in Chinese patients with hepatitis B (43).

The −*3279C*>*A* polymorphism alters the *FOXP3* expression pathway. The ^*^A allele changes the E47 and c-Myb transcription factor binding sites, leading to modifications in the gene expression that predispose the individual to autoimmune disease development (44). In addition, the variant ^*^A interferes with Sp1 transcription factor binding to the *FOXP3* gene, affecting gene expression (45). Findings concerning the impact of the polymorphism on immunological regulation led to new research into its association with other pathologies in different populations, such as recurrent spontaneous abortion in Han Chinese individuals (46), allergic rhinitis in Hungarian individuals (47), and breast cancer tumor progression (48) and preeclampsia (49) in Indian individuals. In cancer cases in Asians, contradictory results have been reported; the ^*^A allele was associated with an increased risk of non-small cell lung cancer (50), while no association between the SNP and breast cancer was found, which led the authors to suggest that the polymorphism may have variable carcinogenic effects in different organs (51).

Studies have shown that the SNP −*924A*>*G* is located in a *FOXP3* gene region equivalent to the GATA3 transcription factor binding site, essential for differentiating the Th2 profile. The presence of the ^*^A allele allows the interaction between GATA3 and *FOXP3* gene, leading to a Th2 profile (46), while ^*^G allele may influence cell conversion (52). The ^*^G allele decreases the expression of the *FOXP3* gene and causes an immunological imbalance, predisposing an individual to developing autoimmune diseases, including an increased risk of developing psoriasis (40) and vitiligo (45).

Due to the lack of associative studies concerning SNPs in the FOXP3 gene with chronic viral liver diseases, the present study breaks new ground due to the acquisition of fundamentally new knowledge concerning immunogenic factors that may interfere with host hepatic function to influence the evolution of pathologies. We believe that these data will provide a basis for future studies regarding the role of FOXP3 as a modulator of the immune response, as well as a better understanding of its function and mechanisms of action in several chronic diseases.

## Materials and methods

### Sample characterization and ethical concerns

This study was performed as a cross-sectional and analytical study in the city of Belém in the state of Pará, Brazil, with the collaboration of the outpatient liver disease clinic of the Hospital of Santa Casa de Misericórdia Foundation of Pará (FSCMPA) and the João de Barros Barreto University Hospital (HUJBB). Consecutive cases of chronic hepatitis B and C carriers were identified and enrolled between 2014 and 2016.

All selected patients were clinically evaluated and subjected to complementary tests based on certain medical criteria, which included enzymatic tests (liver enzyme levels: alanine aminotransferase (ALT), aspartate aminotransferase (AST), and gamma-glutamyl transferase (GGT), serological tests (HBV surface antigen (HBsAg), HBV e antigen (HBeAg), anti-HBeAg, total anti-HBc and anti-HCV), virological tests (HBV DNA and *hepacivirus C* RNA), ultrasound examination, endoscopic tests and liver biopsies, which allowed the patients to be screened and classified as described below.

Forty-one active chronic hepatitis B virus carriers (CHB-A) were identified based on the presence of histological changes in the liver and persistent HBsAg for more than 6 months. Thirty-three inactive chronic hepatitis B virus carriers (CHB-I) were identified based on the absence of significant histological changes, persistently normal liver enzyme levels for at least 1 year, an HBV DNA load below 2,000 copies/mL in blood, and negative HBeAg and positive anti-HBeAg results. One hundred and one chronic hepatitis C carriers (CHC) were identified based on the positive presence of a *hepacivirus C* viral load and changes in the liver histological profile and enzyme levels.

A control group (CG) was established that consisted of 300 volunteer blood donors from the Center for Hemotherapy and Hematology of the Pará Foundation (HEMOPA). The volunteers were seronegative and had undetectable viral loads for HBV, *hepacivirus C* and other agents typically screened for at blood banks.

The inclusion and exclusion criteria in the present study were the same as those established by previous studies published by our research group (53), including an age older than 18 years, provision of consent by the participants through signing an informed consent form and the biological aspects inherent to the study.

In compliance with Resolutions 466/2012 and 347/05 of the National Health Council, which established guidelines and standards for human research in Brazil, the present study was submitted to and approved by the Research Ethics Committee of FSCMPA (protocol #772,782/2014) and HUJBB (protocol 962,537/2015).

### Laboratory data

The liver enzyme levels, serology for the investigated viral liver diseases and plasma viral loads were obtained from updated clinical records when the patients agreed to participate in the study. These data were organized into an access-restricted worksheet used only for updates and for obtaining information related to the study objectives.

### Histopathological procedures

Liver biopsy specimens were obtained only from patients with a medical indication for investigation of hepatic parenchyma alterations within the clinical care protocol. The liver biopsies were performed by medical professionals with a Tru Cut needle (UNIT comércio, importação e exportação LTDA, São Paulo, Brazil) using an ultrasound-guided approach. Each sample was examined at the Pathological Anatomy Service of the Federal University of Pará (UFPA) according to the service's routine protocols. The specimens were stained with hematoxylin and eosin (HE), chromotrope aniline blue (CAB), Gomori's reticulin and Shikata's orcein stains.

The histopathological diagnosis was based on the French METAVIR classification system (54). The activity of the peri-portal and peri-septal inflammatory infiltrates (inflammatory activity) was scored from 0 to 3 (A0-A3), with “A0-A1” indicating absent to mild inflammation and “A2-A3” indicating moderate to severe inflammation. The structural alterations of the hepatic parenchyma (i.e., the degree of fibrosis) were scored from 0 to 4 (F0-F4), with “F0-F1” indicating absent to mild liver fibrosis, “F2” indicating moderate liver fibrosis and “F3-F4” indicating advanced liver fibrosis to cirrhosis. All data regarding the histopathological profiles were obtained from medical records, which were available for data collection.

Patients evaluated by transient elastography (TE) whose results indicated histopathological changes were classified according to the methodology described above without the need for a liver biopsy. However, for these patients, the sensitivity of the testing only permitted the determination of the degree of fibrosis; the inflammatory activity was not evaluated.

### DNA extraction and polymorphism genotyping

For the molecular analyses, a vacuum collection system was used to collect peripheral blood samples directly into a 4-mL collection tube containing EDTA as an anticoagulant. These samples were transported to the Laboratory of Virology of the Federal University of Pará (Labvir-UFPA) and stored at −20°C until subsequent analysis.

DNA was extracted from these samples based on a forensic method consisting of cell lysis, protein precipitation, DNA precipitation and DNA hydration steps, as described in a previous publication (53).

The −*3279C*>*A*, −*2383C*>*T*, and −*924A*>*G* SNPs in the *FOXP3* gene promoter region were genotyped by real-time PCR using a StepOne PLUS Sequence Detector (Applied Biosystems, Foster City, CA, USA) using primers and probes from TaqMan® SNP Genotyping Assays (−***3279***: C_27476877_10, −***2383***: C_27058744_10, and −***924***: C_15942641_10) (Applied Biosystems, Foster City, CA, USA).

For each reaction, 7 μL of distilled water, 10 μL of TaqMan® Universal PCR Master Mix (2X), 1 μL of TaqMan® Assay Buffer (20X) and 2 μL of extracted DNA were used in a final volume of 20 μL. The following thermal cycling program was used to amplify and detect polymorphisms: 60°C for 30 s, followed by 95°C for 10 min, 50 cycles of 92°C for 30 s and 1 cycle at 60°C for 1 min and 30 s.

**Figure 1:**
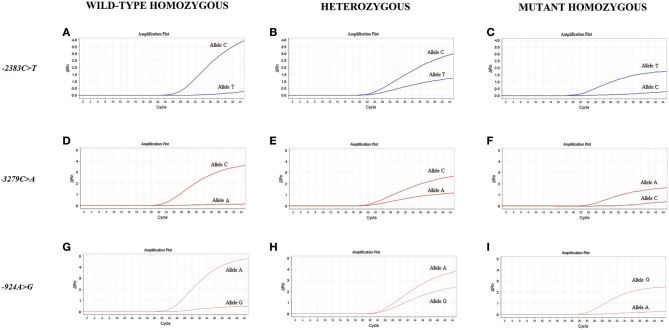
Amplification plots of the genotypes for the studied polymorphisms. The amplification curves are based on the fluorescence intensity variation (ΔRn) according to the number of PCR cycles for the wild-type homozygous **(A,D,G)**, heterozygous **(B,E,H)** and mutant homozygous **(C,F,I)** genotypes of the *FOXP3* polymorphisms.

Figure 1 shows the amplification plots of the different genotypes of the studied polymorphisms.

### Statistical analysis

The genotype and allele frequencies of the polymorphisms were estimated by direct counting, and these frequencies were compared between the study groups using the G and Fisher's exact tests, following the assumptions of each test. Hardy Weinberg equilibrium was estimated using the Chi-squared test. Only females were included in this analysis because the *FOXP3* gene is located on the X chromosome; the same principle was used for the determination of genotype frequencies. The statistical analyses described above were performed using BioEstat 5.0 software (55) at a significance level of 5% (*p* < 0.05).

A haplotype block was inferred from analysis of linkage disequilibrium (LD) between polymorphisms using Haploview 4.2 software (56), assuming a scheme based on the representation of the confidence interval of the normalized linkage disequilibrium coefficient (D'). The Chi-squared test was used to analyze the frequencies of the obtained haplotypes between groups.

The enzymatic and virological data were compared between the polymorphism genotypes with the Mann-Whitney test using BioEstat 5.0 (55) and GraphPad Prism version 6.1 software; the latter was used specifically for estimation of the plots. For significant data, heatmap matrices were plotted using R 3.4.2 software (57) with canberra and ward.D as the distance and cluster methods, respectively, depending on the options provided by the software.

## Results

### Sample description

The majority of patients included in the study were hepatitis C carriers rather than CHB carriers. Male individuals predominated in the CHB-A group. The median viral load values and liver enzyme measurements were higher in CHC patients than in the other groups.

Histopathology profile showed in most cases no signs of abnormalities (with the inflammatory activity and hepatic fibrosis mostly absent to mild) with the exception of CHB-A carriers.

Eleven CHC and eight CHB-A patients were not submitted to biopsy considering the high risk procedure as the previous results of TE indicated severe alterations in the hepatic parenchyma; these patients were classified according to the degree of fibrosis.

The data on sex, viral load, and liver enzyme levels, inflammatory activity and degree of hepatic fibrosis are shown in Table 1.

**Table 1:** Laboratory and histopathological data for patients with chronic hepatitis B and C virus infections.

**Variables**	**CHC**	**CHB-I**	**CHB-A**
Number of individuals	101	33	41
Sex (*F/M*)	50/51	16/17	12/29
Viral load (log*^10^*) mean ±σ	5.41 ± 1.02	2.27 ± 0.78	3.74 ± 1.58
Median	5.62	2.03	3.90
ALT (IU/L) mean ±σ	77.93 ± 58.42	27.52 ± 14.83	80.05 ± 101.19
Median	58.00	31.00	44.00
AST (IU/L) mean ±σ	69.83 ± 48.62	29.82 ± 14.56	60.98 ± 54.73
Median	59.00	25.00	40.00
GGT (IU/L) mean ±σ	99.58 ± 95.18	31.79 ± 21.22	64.35 ± 28.53
Median	60.00	25.00	36.00
**DEGREE OF INFLAMMATORY ACTIVITY**			
Absent to mild (%)	40 (58.82)	10 (100.00)	16 (76.19)
Moderate to severe (%)	28 (41.18)	–	5 (23.81)
**DEGREE OF LIVER FIBROSIS**			
Absent to mild (%)	27 (34.18)	10 (100.00)	10 (34.48)
With few septa (%)	17 (21.52)	–	9 (31.04)
Advanced to cirrhosis (%)	35 (44.30)	–	10 (34.48)

*σ, Standard deviation*.

### Allele and genotype frequencies of *Foxp3* gene polymorphisms

Table 2 shows the allele and genotype frequencies of the studied polymorphisms in the *FOXP3* gene organized according to the infection status and the sex of the participants. All polymorphisms were in Hardy Weinberg equilibrium for the analyzed groups.

**Table 2:** Genotype and allele polymorphism frequencies in the *FOXP3* gene promoter region in patients with chronic hepatitis B and C virus infections.

**Genotypic and allelic profile**	**CHC**	**CHB**		**CG**	***p1***	***p2***	***p3***	***p4***	***p5***	***p6***
		**I**	**A**							
	***n*** **(%)**	***n*** **(%)**	***n*** **(%)**	***n*** **(%)**						
**FEMALES**^#^										
−*2383C*>*T*										
*CC*	41 (82.00)	12 (80.00)	9 (81.82)	133 (89.26)	1.0000	1.0000	1.0000	1.0000	1.0000	1.0000
*CT*	9 (18.00)	3 (20.00)	2 (18.18)	16 (10.74)						
*TT*	0	0	0	0						
^*^C	0.910	0,900	0.909	0.946	0.2324	0.9731	0.9938	0.3985	1.0000	0.6185
^*^T	0.090	0.100	0.091	0.054						
−*3279C*>*A*										
*CC*	27 (54.00)	13 (81.25)	8 (66.67)	91 (61.07)	0.4961	0.4913	0.1179	0.2500	0.3555	0.2500
*AC*	20 (40.00)	2 (12.50)	4 (33.33)	46 (30.87)						
*AA*	3 (06.00)	1 (06.25)	0	12 (08.05)						
^*^C	0.740	0.875	0.833	0.765	0.6854	0.4322	0.1475	0.6152	0.7131	0.1859
^*^A	0.260	0.125	0.167	0.235						
−*924A>G*										
*AA*	11 (22.00)	3 (18.75)	1 (09.09)	46 (30.87)	0.4709	0.2337	0.8526	0.1034	0.2507	0.4749
*AG*	27 (54.00)	8 (50.00)	9 (81.82)	73 (48.99)						
*GG*	12 (24.00)	5 (31.25)	1 (09.09)	30 (20.13)						
^*^A	0,490	0,438	0.500	0.554	0.2975	1.0000	0.6861	0.6618	0.7827	0.2629
^*^G	0.510	0.563	0.500	0.466						
MALES^*^										
−*2383C>T*										
*C*	48 (94.12)	13 (81.25)	24 (92,31)	138 (91.39)	0.7655	0.9967	0.1424	1.0000	0.3520	0.3692
*T*	3 (05.88)	3 (18.75)	2 (07.69)	13 (08,61)						
−*3279C>A*										
*C*	45 (88.24)	13 (76.47)	17 (65.39)	112 (74.17)	0.0503	0.0305	0.2535	0.4739	0.5131	1.0000
*A*	6 (11.76)	4 (23.53)	9 (34.61)	39 (25.83)						
−*924A>G*										
*A*	31 (60.79)	7 (41.18)	10 (65.52)	82 (54.31)	0.5144	0.0358	0.2591	0.0674	0.7553	0.3196
*G*	20 (39.22)	10 (58.83)	19 (34.48)	69 (45.69)						

p1, CHC vs. GC; p2, CHC vs. CHB-A; p3, CHC vs. CHB-I; p4, CHB-A vs. GC; p5, CHB-A vs. CHB-I; p6, CHB-I vs. GC;

# G Test;

* Fisher's exact test.

For the −*2383C*>*T* polymorphism, the ^*^C allele was the most frequent among the studied groups and both sexes; however, no significant differences were observed between the genotype and allele frequencies of the variants of this polymorphism. The frequency of the ^*^C allelic variant of the −*3279C*>*A* polymorphism was significantly higher in the male patients in the CHC group than in the CHB-A group (*p* = 0.0305). A similar trend was observed between the CHC and CG groups, but the significance value in this case was borderline (*p* = 0.0503). For the −*924A*>*G* polymorphism, the frequency of the ^*^A allele was significantly higher in male patients in the CHB-A group than in the CHC group (*p* = 0.0358).

**Figure 2:**
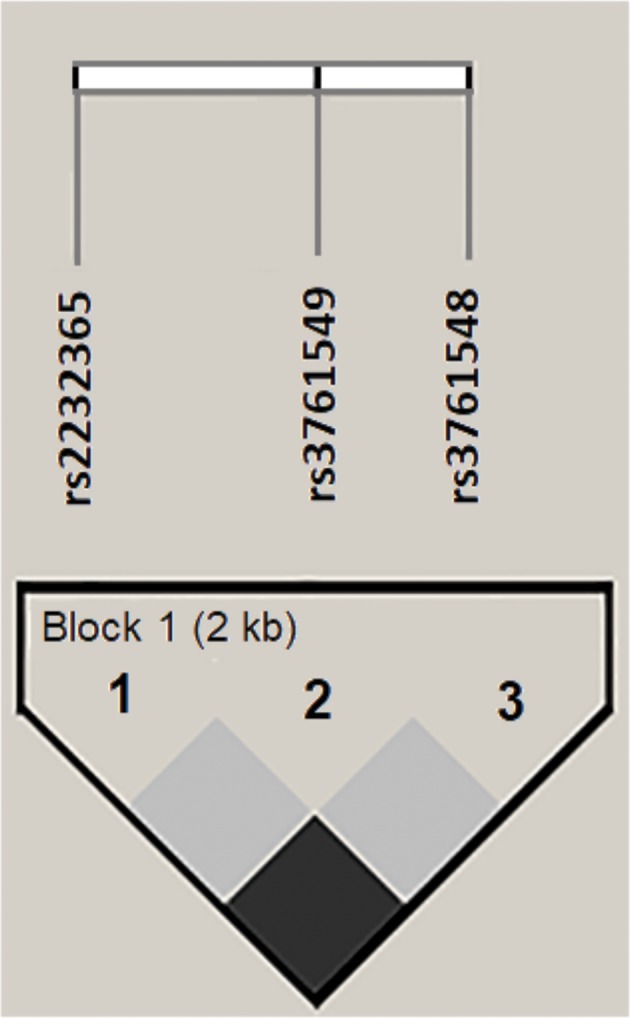
Linkage disequilibrium (LD) for polymorphisms in the *FOXP3* gene promoter region generated in Haploview 4.2. The horizontal white bar illustrates the physical arrangement of polymorphisms along the gene. The normalized linkage disequilibrium coefficients (D') were equal to 1 for all combinatorial analyses. A 2-kb haplotype block was inferred in the analysis.

A haplotype block for the analyzed polymorphisms was inferred (Figure 2). It was observed that the *ACC* haplotype prevailed in all groups analyzed, and the *ATC* and *GTA* haplotypes were found only in the CHB-I group. However, no significant differences were found in the comparison of haplotype frequencies between the studied groups. The haplotypes and their respective frequencies are shown in Table 3.

**Table 3:** Haplotype frequencies of polymorphisms of the *FOXP3* gene promoter region (−*924A*>*G*,−*2383C*>*T*, and −*3279C*>*A*) in patients with chronic hepatitis B and C virus infections.

**Haplotypes**	**CHC**	**CHB**	**CG**	***p1***	***p2***	***p3***	***p4***	***p5***	***p6***	
		**I**	**A**							
	***n*** **(%)**	***n*** **(%)**	***n*** **(%)**	***n*** **(%)**						
**BLOCK 1**										
*ACC*	69 (0.550)	15 (0.400)	14 (0.383)	201 (0.548)	1.0000	1.0000	0.2015	1.0000	0.6837	0.0728
*CGA*	29 (0.188)	5 (0.150)	8 (0.267)	97 (0.247)						
*CGC*	27 (0.188)	12 (0.300)	9 (0.250)	60 (0.135)						
*GTC*	12 (0.074)	4 (0.100)	4 (0.100)	29 (0.070)						
*ATC*	–	1 (0.017)	–	–						
*GTA*	–	1 (0.017)	–	–						

*p1, CHC vs. GC; p2, CHC vs. CHB-A; p3, CHC vs. CHB-I; p4, CHB-A vs. GC; p5, CHB-A vs. CHB-I; p6, CHB-I vs. GC*.

### Analysis of polymorphisms in terms of liver function and histopathological aspects

The distributions of the genotype frequencies of the polymorphisms based on the degrees of inflammatory activity and liver fibrosis are shown in Table 4.

**Table 4:** Frequency of polymorphisms in the *FOXP3* gene promoter region according to the histopathological characteristics of patients with chronic hepatitis B and C virus infections.

**Infection and genetic profile**	**Degree of inflammatory activity**			**Degree of liver fibrosis**					
	**Absent to mild**	**Moderate to severe**	***p1***	**Absent to mild**	**With few septa**	**Advanced to cirrhosis**	***p2***	***p3***	***p4***
	***n*** **(%)**	***n*** **(%)**		***n*** **(%)**	***n*** **(%)**	***n*** **(%)**			
−*2383C>T*									
CHC									
*CC*	35 (87.50)	26 (92.86)	0.6147	24 (88.89)	16 (94.12)	32 (91.43)	0.6808	0.5221	1.0000
*CT*	4 (10.00)	2 (07.14)		2 (07.41)	1 (05.88)	3 (08.57)			
*TT*	1 (02.50)	0		1 (03.71)	0	0			
CHB-I									
*CC*	6 (66.67)	–	–	6 (66.67)	–	–	–	–	–
*CT*	0	–		0	–	–			
*TT*	3 (33.33)	–		3 (33.33)	–	–			
CHB-A									
*CC*	13 (86.67)	5 (100.00)	0.7014	8 (88.89)	8 (88.89)	8 (100.00)	0.3973	1.0000	1.0000
*CT*	1 (06.67)	0		1 (11.11)	0	0			
*TT*	1 (06.67)	0		0	1 (11.11)	0			
−*3279C>A*									
CHC									
*CC*	31 (77.50)	20 (71.42)	0.6840	22 (81.48)	11 (64.70)	27 (77.14)	0.3043	0.7484	0.6046
*AC*	6 (15.00)	4 (14.29)		4 (14.81)	3 (17.65)	5 (14.29)			
*AA*	3 (07.50)	4 (14.29)		1 (03.70)	3 (17.65)	3 (08.57)			
CHB-I									
*CC*	7 (77.78)	–	–	7 (77.78)	–	–	–	–	–
*AC*	1 (11.11)	–	–	1 (11.11)	–	–			
*AA*	2 (22.22)	–	–	2 (22.22)	–	–			
CHB-A									
*CC*	10 (66.67)	4 (80.00)	0.3131	5 (50.00)	7 (87.50)	6 (66.67)	0.0943	0.5462	0.1253
*AC*	1 (06.67)	1 (20.00)		1 (10.00)	1 (12.50)	0			
*AA*	4 (26.66)	0		4 (40.00)	0	3 (33.33)			
−*924A>G*									
CHC									
*AA*	18 (45.00)	13 (46.43)	0.9920	9 (33.33)	9 (52.94)	19 (54.29)	0.4320	0.2669	0.9562
*AG*	9 (22.50)	6 (21.43)		8 (29.63)	3 (17.65)	7 (20.00)			
*GG*	13 (32.50)	9 (32.14)		10 (37.04)	5 (29.41)	9 (25.71)			
CHB-I									
*AA*	3 (30.00)	–	–	3 (30.00)	–	–	–	–	–
*AG*	1 (10.00)	–		1 (10.00)	–	–			
*GG*	6 (60.00)	–		6 (60.00)	–	–			
CHB-A									
*AA*	4 (25.00)	2 (40.00)	0.8404	1 (10.00)	5 (55.56)	2 (20.00)	0.1108	0.7890	0.3130
*AG*	4 (25.00)	1 (20.00)		3 (30.00)	1 (11.11)	2 (20.00)			
*GG*	8 (50.00)	2 (40.00)		6 (60.00)	3 (33.33)	6 (60.00)			

*p1, Inflammation absent to mild vs. moderate to severe; p2, Fibrosis absent to mild vs. with few septa; p3, Fibrosis absent to mild vs. advanced to cirrhosis; p4, Fibrosis with few septa vs. advanced to cirrhosis*.

The *CC* genotype of the −*2383C*>*T* polymorphism was the most frequent among the study groups when analyzing both the inflammatory activity and the degree of liver fibrosis. Similarly, for the −*3279C*>*A* polymorphism, the *CC* genotype was the most frequent under these same conditions. However, significant differences were not identified in the comparison of the frequencies of these polymorphisms between the different histopathological features for the analyzed groups.

For the −*924A*>*G* polymorphism, the *AA* genotype was the most frequent in the CHC group regardless of the degree of inflammatory activity; however, the *GG* genotype became the most frequent in this group when the liver fibrosis was absent to mild. The *GG* genotype was also the most frequent in the CHB-I group, regardless of histopathological stratification; it was also the most frequent in the CHB-A group, except in patients with liver fibrosis with few septa, for whom the *AA* genotype was the most frequent. However, no significant differences were observed in the frequencies of genotypic variants of this polymorphism according to the histopathological findings in the analyzed groups.

For SNP −*2383C*>*T*, no significant differences were found in the comparison of enzymatic and virological markers with the degrees of inflammatory activity and liver fibrosis (Supplementary Figures 1–4).

**Figure 3:**
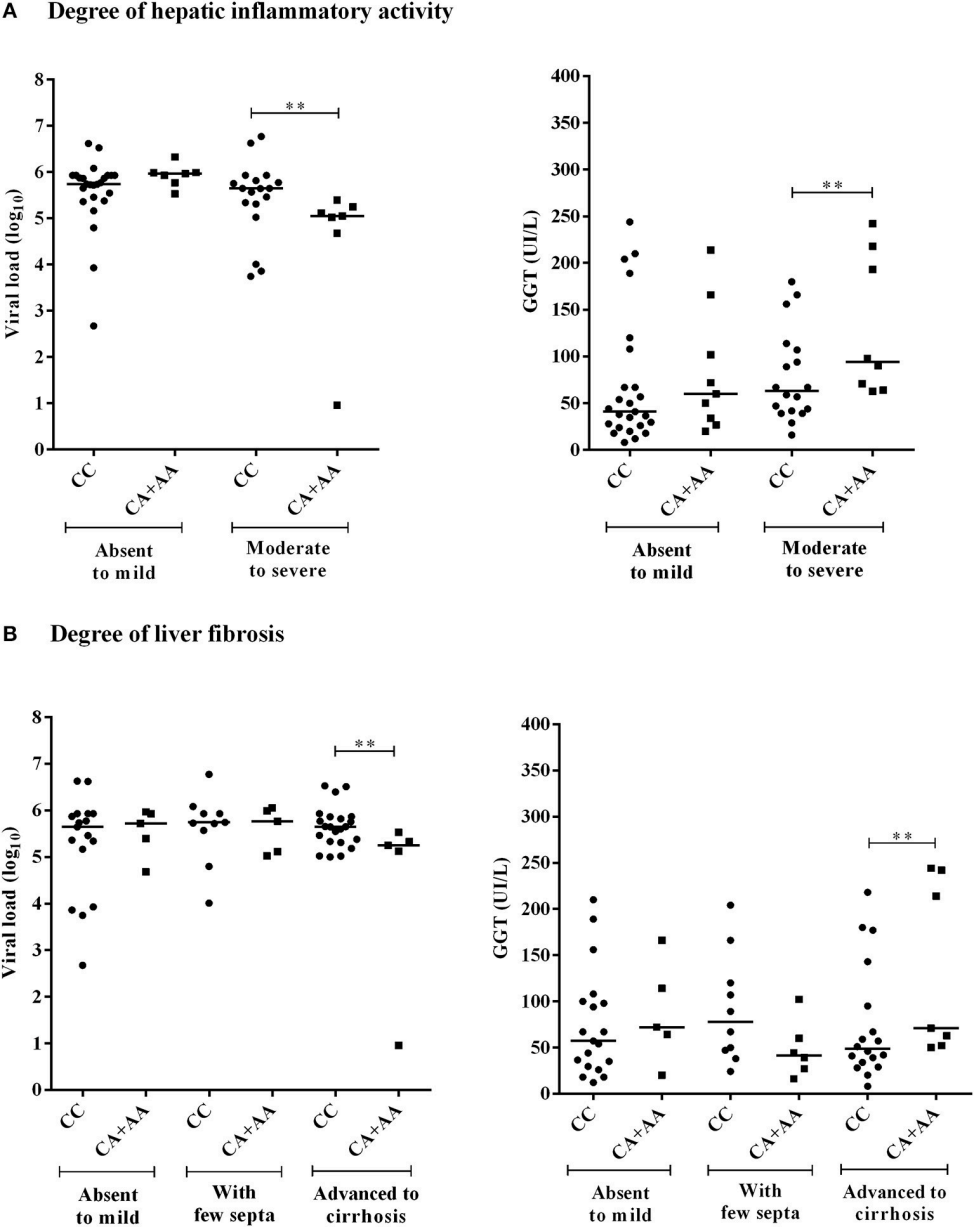
Comparison of the plasma viral loads (log_10_) and GGT levels among CHC patients with −*3279C*>*A* polymorphism genotypes according to the degree of inflammatory activity **(A)** and liver fibrosis **(B)**. Significant changes in markers were observed at the more severe histopathological stages (**).

**Figure 4:**
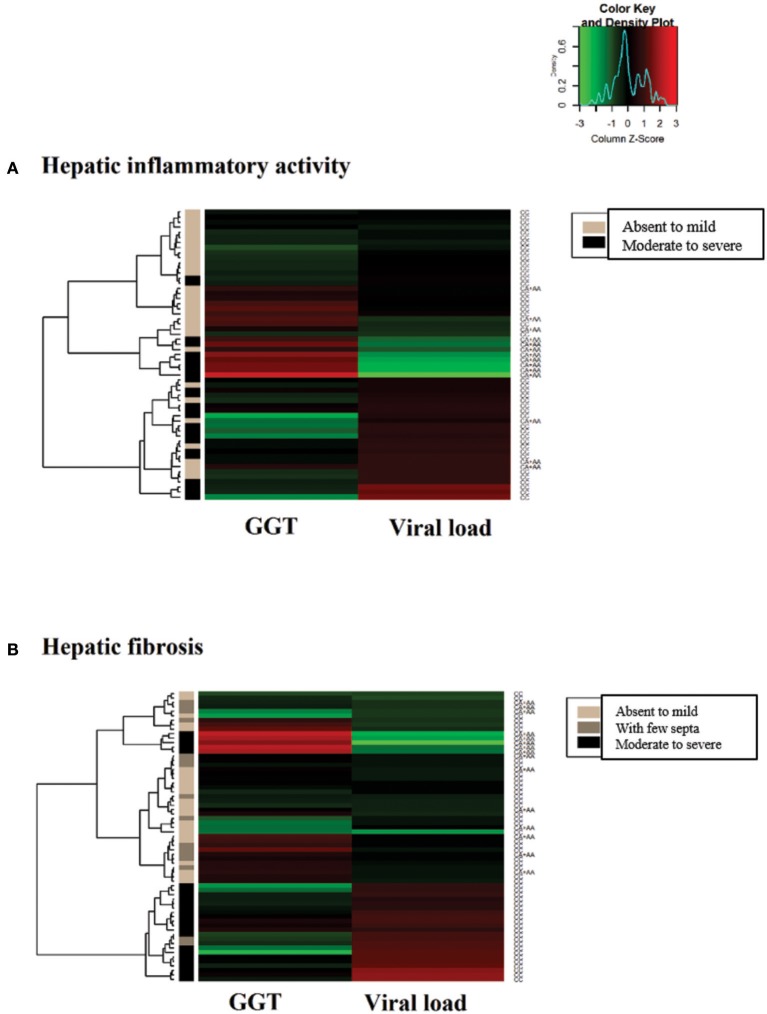
Heatmap matrix analysis of liver function profiles according to the histopathological characteristics and −*3279C*>*A* polymorphism genotypes in CHC patients. **(A)** Inflammatory activity and high viral loads clustered with low to intermediate GGT levels in a group composed predominantly of individuals with the *CC* genotype and with moderate to severe inflammatory activity. Sub-cluster groups with high GGT levels and low viral loads in individuals with the *A allele in homozygous or heterozygous individuals with moderate to severe inflammatory activity. **(B)** In patients with liver fibrosis, a cluster was formed with high viral loads and low to intermediate GGT levels predominantly in individuals with the CC genotype and advanced liver fibrosis to cirrhosis.

**Figure 5:**
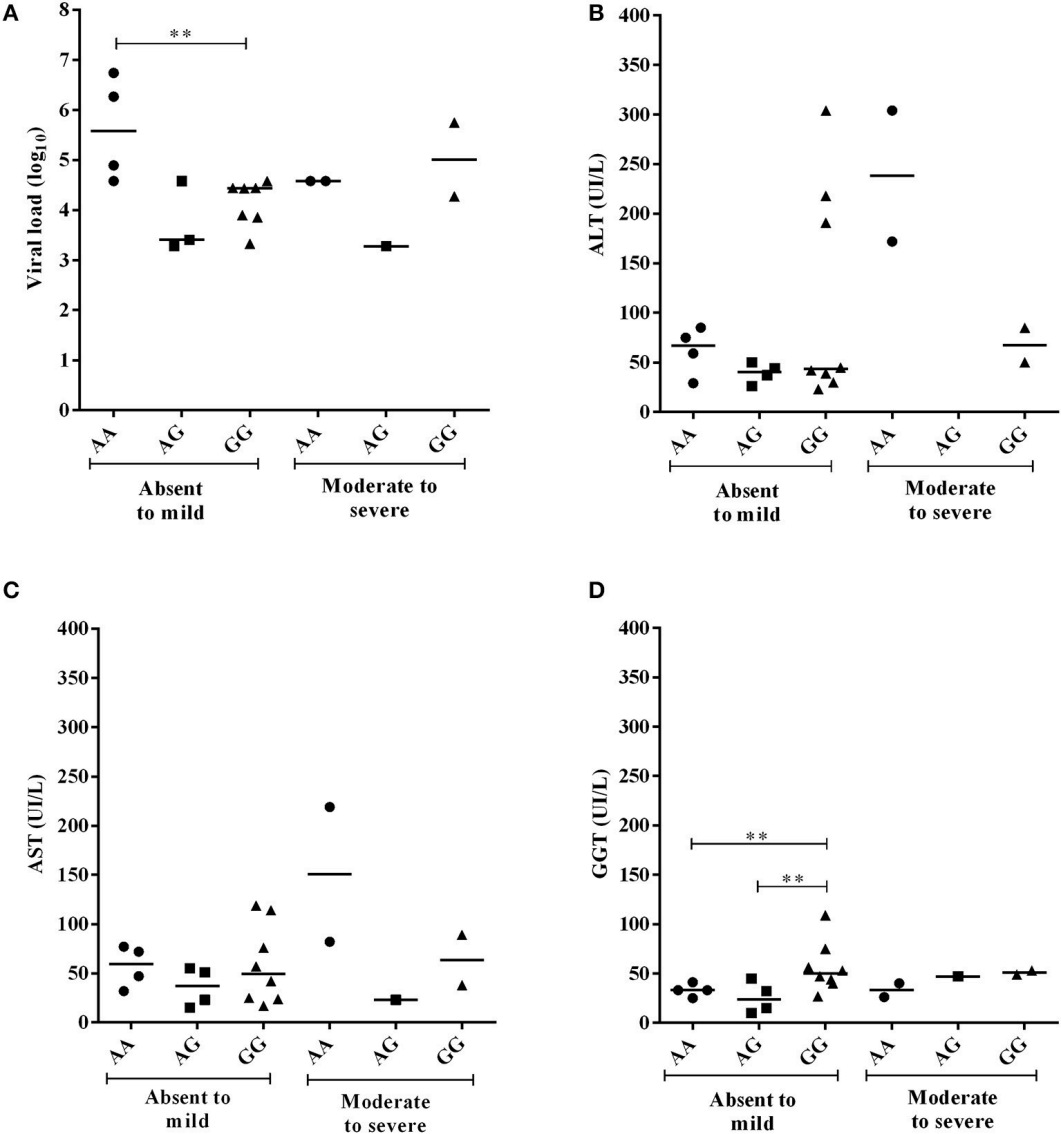
Comparison of plasma viral loads (log_10_) and liver enzyme levels in individuals with −*924A*>*G* polymorphism genotypes in CHB-A patients according to the degree of inflammatory activity. Statistical significance was observed for the plasma viral loads (log_10_) **(A)** and GGT levels **(B)** at the milder stages of tissue inflammation (**), but not to AST and ALT enzymes (**C,D**).

**Figure 6:**
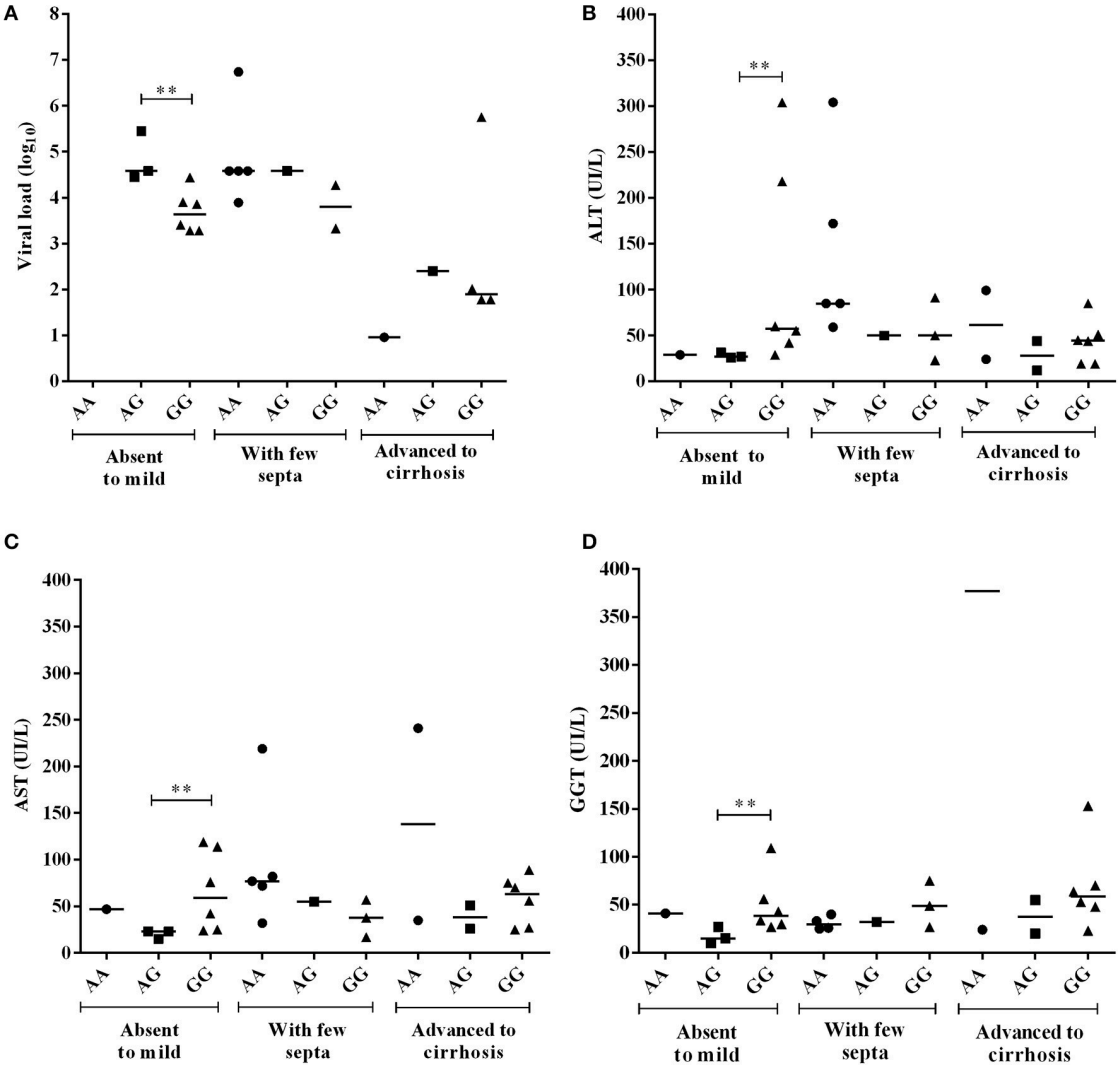
Comparison of plasma viral loads (log_10_) and liver enzyme levels among CHB-A patients with −*924A*>*G* polymorphism genotypes according to the degree of liver fibrosis. All comparisons were significant **(A–D)** at the absent to mild fibrosis stages (**).

**Figure 7:**
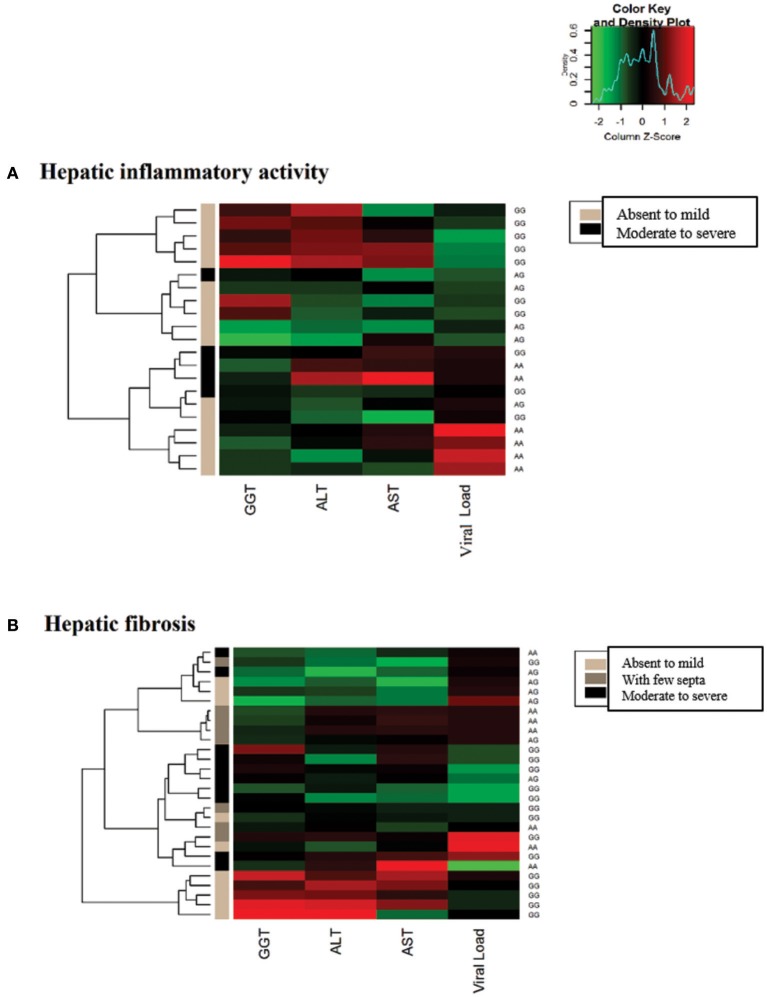
Heatmap matrix constructed to analyze the liver function profiles according to the histopathological characteristics and −*924A*>*G* polymorphism genotypes in CHB-A patients. **(A)** Regarding inflammatory activity, high hepatic enzyme levels were clustered with low viral loads for patients with the *GG* genotype with mild to absent inflammatory activity. Another cluster was formed among patients with low hepatic enzyme levels and high viral loads in *AA* genotype individuals with low tissue inflammation. **(B)** Regarding liver fibrosis, elevated liver enzyme levels were clustered with intermediate viral loads for *GG* genotype patients with mild to absent fibrosis.

Figures 3–**7** refer to comparative analyses of enzymatic markers of liver function and plasma viral loads (log_10_) between the genotypes of the −*3279C*>*A* and −*924A*>*G* polymorphisms.

The viral loads were lower in CHC patients with moderate to severe inflammation who were homozygous or heterozygous for the ^*^A allele of the −*3279C*>*A* polymorphism (*p* = 0.0199). Conversely, the GGT levels were higher for these patients (*p* = 0.0454) than for carriers of the *CC* genotype (Figure 3A). This result was similar to observations made in the analysis of these markers for the advanced liver fibrosis to cirrhosis stages (viral load, *p* = 0.0261; GGT, *p* = 0.0405) (Figure 3B).

In the heatmap matrix plotted to analyze the −*3279C*>*A* polymorphism in relation to the histopathological features in the CHC group (Figure 4), the highest viral loads in patients with the *CC* genotype with moderate to severe inflammation were predominantly clustered together with low and intermediate GGT levels. The plotted sub-clusters showed an inverse relationship; however, complementary to previously established findings, high GGT levels clustered with low viral loads in carriers of the ^*^A allele with moderate to severe inflammatory activity (Figure 4A), which was consistent with established statistical data.

A similar trend was observed for liver fibrosis. High viral load levels were grouped with low GGT levels in patients with the *CC* genotype in a cluster predominantly formed by patients with advanced fibrosis to cirrhosis (Figure 4B).

For the −*924A*>*G* polymorphism, CHB-A patients with the *GG* genotype and absent to mild inflammatory activity had lower viral loads than patients with the *AA* genotype (*p* = 0.0121), and higher GGT levels than patients with both the *AG* genotype (*p* = 0.0485) and the *AA* genotype (*p* = 0.0465) (Figure 5). In the liver fibrosis analysis, patients with mild fibrosis with the *GG* genotype had lower viral loads (*p* = 0.0238) but higher ALT (*p* = 0.0476), AST (*p* = 0.0238) and GGT levels (*p* = 0.0357) than patients with the *AG* genotype (Figure 6).

In the heatmap for the −*924A*>*G* polymorphism in patients in the CHB-A group with inflammatory activity, lower viral loads were clustered with high liver enzyme levels in patients with absent to mild inflammation with the *GG* genotype. Additionally, the highest viral loads tended to cluster with low and intermediate levels of liver enzymes in patients with absent to mild inflammation with the *AA* genotype. In intermediate groups, whose low levels of hepatic enzymes and viral loads were grouped, the prevalence of heterozygous genotypes is likely to be a contributing factor (Figure 7A).

In the heatmap matrix of liver fibrosis, the trends established for the −*924A*>*G* polymorphism in the CHB-A group were clearly visualized. In the group of patients with the *GG* genotype and absent to mild fibrosis, the highest levels of liver enzymes were clustered with intermediate viral loads (Figure 7B), similar to the obtained statistical data.

For the CHB-I patients, analyses of the liver function and virological variables did not show any significant differences in relation to the histopathological features and the genotypic profiles of the studied polymorphisms (data not shown).

## Discussion

Polymorphisms in the *FOXP3* gene promoter region are notable for their ability to alter gene expression of the transcription factor and consequently modify the natural course of Treg cell activation (36). Thus, conducting studies to evaluate the characteristics of these genetic variations in different contexts is important, with the goal of producing and/or substantiating knowledge of disease-related immunogenetic interactions.

The ^*^T variant −*2383C*>*T* was associated with increased FOXP3 expression in the present study; we expected that its frequency would be high in the group with viral hepatopathy. Higher *FOXP3* levels may lead to an intense regulatory response in the liver, resulting in a decline in the virus-specific immune response and viral recrudescence, which prevents collateral damage induced by excessive immune activation but may be a factor leading to the maintenance of chronic infection (15, 58, 59). However, we observed that the genotype and allele frequencies of the ^*^C variant were most prevalent among the analyzed groups, which also had repercussions for the sequences of the obtained haplotypes. In addition, no significant differences were identified between the genetic variants relative to the virological, histopathological and liver function characteristics, suggesting that this polymorphism might not directly influence the modulation of the immune response and/or hepatic immunotolerance via FOXP3-dependent pathways in patients with chronic viral liver diseases.

Although Chen et al. identified an association of the *CT* and *TT* genotypes with the recurrence of HBV-mediated hepatocellular carcinoma, the authors suggested a closer investigation into the incidence of tumors and its relation to the polymorphism was necessary to confirm if this relationship was due to the infection or carcinoma itself (43). Based on data from the present study, the polymorphic variants of this SNP are not determinants for the establishment of infection or its progression, since associations with aspects of chronic hepatitis B were not confirmed or established. We propose that subsequent studies evaluate the role of this polymorphism in hepatocarcinogenesis, since it has been associated with susceptibility to other cancer types (60). Furthermore, in a carcinogenic microenvironment, Treg expansion favors the maintenance of anti-cancer immunity (61–63), which may explain the possible effects of this polymorphism.

In the present study, the ^*^C allele of the −*3279C*>*A* polymorphism was significantly more prevalent in patients with chronic hepatitis C infection. In fact, previous studies have indicated that this polymorphism is a genetic factor that predisposes patients to susceptibility and/or progression of certain pathological conditions by favoring *FOXP3* gene expression (44, 45, 48, 64–66). Therefore, if the ^*^C variant maintains *FOXP3* gene expression, this variant may participate in the immunopathogenesis of chronic hepatitis C infection, likely through activation of intrahepatic Tregs. This possibility may explain why these cells are abundant in lymphocytic infiltrates in the portal space and hepatic lobes (58).

This finding was in agreement with the low viral loads and high GGT levels in ^*^A allele carriers based strictly on the high degrees of inflammation and fibrosis represented by the clusters inferred in the heatmap matrices. If the polymorphism limits the activation and/or expansion of Tregs, a decrease in the suppressive function of these cells and an imbalance between liver damage and control of viral replication are likely to occur (67–69). Thus, in individuals with the ^*^A allele, these possible alterations in the regulatory response may induce maintenance of a cytolytic inflammatory response capable of leading to a decreased viral load and increased tissue damage in the hepatic microenvironment. The association with the GGT enzyme further supports the identified results, because it is a marker clinically employed in the analysis of liver fibrosis, especially when there is blockage of the bile ducts and intense necroinflammatory activity (70–73).

The literature also provides data that correlate *FOXP3* expression and the presence of Treg cells with higher levels of inflammatory activity and the degree of liver fibrosis in chronic viral hepatic diseases (74–76). Thus, in patients with chronic hepatitis C infection, the −*3279C*>*A* polymorphism may mediate Treg activation in more advanced stages of liver inflammation and fibrosis, as shown by the relationships established in the present study. However, since the frequency of polymorphic variants was not associated with the degrees of inflammatory activity and liver fibrosis, we propose that this polymorphism may be influencing certain aspects of immune modulation during chronic hepatitis C infection but is not directly related to establishment of the histopathological scores *per se*. This proposal emphasizes the multifactorial nature of the pathological condition and the evolution of liver damage, which is not restricted to only the host-parasite relationship but also influenced by metabolic, physiological and toxicity exposure conditions (77).

For the −*924A*>*G* polymorphism, the frequency of the ^*^G allele was significantly lower in the CHB-A group than in patients with chronic hepatitis C infection. Simultaneously, patients with the *GG* genotype had lower viral loads and higher hepatic enzyme levels in the milder stages of the histopathological characteristics of the disease, as represented in the clusters plotted in the heatmaps. Changes in the balance of the Th1 and Th2 profiles generated by the allelic variants of this polymorphism have been proposed to be fundamental for the maintenance of certain pathological conditions (40, 45, 65) and may also influence chronic hepatitis B carrier status based on the data obtained in the present study.

In active hepatitis B virus infection, the Treg-Th2 conversion induced by the ^*^A variant most likely contributes to the development of a proinflammatory Th1 response in hepatic tissue. In fact, Th2 induction favors the persistence of infection due to stimulation of the production of anti-inflammatory cytokines, such as IL-4 and IL-10 (78), which can reduce tissue damage and decrease inflammatory activity, as proposed by the data reported in the present study.

Conversely, in the absence of Th2-specific signaling, FOXP3+ Treg cells can express high levels of IFN-γ and T-bet, which are Th1-specific transcription factors, making them susceptible to conversion to the Th1 profile (79, 80). Reportedly, blockage of the Treg-Th2 conversion generated by the ^*^G allele of the −*924A*>*G* polymorphism (76) can promote an immune balance favorable to the development of a Th1 profile, which is considered crucial for the control of viral replication by tissue inflammation (81, 82). This situation would lead to tissue damage, with a subsequent increase in liver enzyme levels in the blood of these patients.

The relationship established between the −*924A*>*G* polymorphism and enzymatic and virological characteristics in the early stages of inflammation and liver fibrosis indicates that the gene activation pathway for this polymorphism may be relevant in maintaining mild tissue injury during active hepatitis B infection. This possibility could complement the proposed negative correlation between the frequency and function of Treg cells in chronic hepatitis B patients with fibrosis and necroinflammatory activity scores (74, 83). However, similar to the −*3279C*>*A* polymorphism, the frequency of the −*924A*>*G* variant was not associated with the histopathological characteristic of patients with active hepatitis B. Therefore, these variants may influence the immunopathogenesis of the infection but are likely not directly related to the establishment of the degree of inflammation or tissue damage.

The results of this study highlight the importance of FOXP3 as a modulating factor of viral liver diseases, as polymorphic variations in the *FOXP3* gene can influence the pathology associated with hepatic infection. Indeed, FOXP3 is not expected to have a consistent positive association between genetic variation and a pathological condition in all study populations, which can be exacerbated by differences between ethnic populations (84). The relative risk of a variant associated with a disease is maintained among different ethnic groups if it is a true susceptibility locus. Polymorphisms in the *FOXP3* promoter region have been associated with different pathological features despite differences in some variant frequencies among different ethnic groups. This finding supports the hypothesis that genetic variations in *FOXP3* represent true susceptibility loci and are not solely variants in linkage disequilibrium with the true locus, as has been observed in certain populations due to the structure of the ancestral haplotype (85).

## Conclusion

Based on the data reported in this study, we conclude that the −*924A*>*G* and −*3279C*>*A* polymorphisms in the *FOXP3* gene promoter region alter viral loads and liver enzyme levels in patients with chronic viral liver diseases. The interference of these polymorphisms varies according to the histopathological stage of the hepatic tissue, although they are not direct determinants of the establishment of histopathological characteristics. In summary, the data presented here further scientific knowledge regarding the role of the *FOXP3* gene and its genetic variations in immunological regulation that determines the establishment and/or progression of pathological conditions. Further studies must be conducted to expand these analyses in the context of infectious diseases.

## Author contributions

AV, SdSC, and RI conceived and designed the experiments. LP and AV wrote the paper. RM-F, AdS, and JM assisted with editing the paper; LP, SdSC, EA, and SD performed the experiments. AV, LP, AdS, and EA analyzed the data. AV, SD, and RI contributed reagents, materials, and analysis tools.

### Conflict of interest statement

The authors declare that the research was conducted in the absence of any commercial or financial relationships that could be construed as a potential conflict of interest.

## Supplementary material

Supplementary Figure 1 Comparison of plasma viral loads (log _10_) and liver enzyme levels (ALT, AST, and GGT) among −*2383C*>T polymorphism genotype individuals in CHC patients with absent to mild and moderate to severe inflammatory activity. Reference values: ALT (8-54 IU/L), AST (16-40 IU/L), and GGT (8-63 IU/L).

Supplementary Figure 2 Comparison of plasma viral loads (log _10_) and liver enzyme levels (ALT, AST, and GGT) among −*2383C*>T polymorphism genotype individuals gene in CHC patients with absent to mild liver fibrosis, fibrosis with few septa and advanced fibrosis to cirrhosis. Reference values: ALT (8-54 IU/L), AST (16-40 IU/L), and GGT (8-63 IU/L).

Supplementary Figure 3 Comparison of plasma viral loads (log _10_) and liver enzyme levels (ALT, AST, and GGT) among −*2383C*>T polymorphism genotype individuals in CHB patients with absent to mild and moderate to severe inflammatory activity. Reference values: ALT (8-54 IU/L), AST (16-40 IU/L), and GGT (8-63 IU/L).

Supplementary Figure 4 Comparison of plasma viral loads (log _10_) and liver enzyme levels (ALT, AST and GGT) among −*2383C*>T polymorphism genotype individuals in CHB patients with absent to mild liver fibrosis, fibrosis with few septa and advanced fibrosis to cirrhosis. Reference values: ALT (8-54 IU/L), AST (16-40 IU/L) and GGT (8-63 IU/L).

## Abbreviations

ALT: alanine aminotransferase
AST: aspartate aminotransferase
FOXP3: Forkhead box P3
GGT: gamma-glutamyl transpeptidase
HBV: hepatitis B virus
CHB-A: active chronic hepatitis B carriers
CHB-I: inactive chronic hepatitis B carriers
CHC: chronic hepatitis C carriers
SNPs: single nucleotide polymorphisms
Tregs: regulatory T lymphocytes.
